# BISON: bio-interface for the semi-global analysis of network patterns

**DOI:** 10.1186/1751-0473-1-8

**Published:** 2006-11-29

**Authors:** Christopher Besemann, Anne Denton, Nathan J Carr, Birgit M Prüβ

**Affiliations:** 1 Department of Computer Sciences, North Dakota State University, Fargo ND 58105, USA; 2 Department of Veterinary and Microbiological Sciences, North Dakota State University, Fargo ND 58105, USA

## Abstract

**Background:**

The large amount of genomics data that have accumulated over the past decade require extensive data mining. However, the global nature of data mining, which includes pattern mining, poses difficulties for users who want to study specific questions in a more local environment. This creates a need for techniques that allow a localized analysis of globally determined patterns.

**Results:**

We developed a tool that determines and evaluates global patterns based on protein property and network information, while providing all the benefits of a perspective that is targeted at biologist users with specific goals and interests. Our tool uses our own data mining techniques, integrated into current visualization and navigation techniques. The functionality of the tool is discussed in the context of the transcriptional network of regulation in the enteric bacterium *Escherichia coli*. Two biological questions were asked: (i) Which functional categories of proteins (identified by hidden Markov models) are regulated by a regulator with a specific domain? (ii) Which regulators are involved in the regulation of proteins that contain a common hidden Markov model? Using these examples, we explain the gene-centered and pattern-centered analysis that the tool permits.

**Conclusion:**

In summary, we have a tool that can be used for a wide variety of applications in biology, medicine, or agriculture. The pattern mining engine is global in the way that patterns are determined across the entire network. The tool still permits a localized analysis for users who want to analyze a subportion of the total network. We have named the tool BISON (Bio-Interface for the Semi-global analysis Of Network patterns).

## Background

Research on biological networks is a well-established part of bioinformatics [1]. Examples of biological networks include regulatory networks [2], protein-protein interactions [3,4], and domain-fusion networks [5,6], among others. Typical objectives are to gain information about the over-all structure and evolution of the network in question [7,8]. Protein function and other annotations are rarely included in network studies and, if so, the results are normally limited to the statistics of similarity or dissimilarity between neighbors [1] or a correlation of function with traditional subgraph statistics [9]. Note that in the following, we will use the terms network and graph interchangeably. We will sometimes refer to proteins in a network as nodes of a graph, and to regulatory or other interactions as edges.

The large amount of annotation and network data that has accumulated over the past decades requires the use of data mining techniques. Pattern mining is a subset of data mining that has the goal of identifying frequently occurring combinations of items of information. We will refer to pieces of information, such as domain and functional information, as properties. Initial work considered simple types of item information [10,11]. Pattern mining techniques have also been used to find frequent subgraphs of larger graphs [12,13]. The most general case of pattern mining considers any combination of relational tables [14]. Recently, the specific problem of finding patterns that involve networks and item data has gained importance [15-17].

BISON integrates our own pattern mining techniques with modern graph visualization and navigation techniques. Combinations of visualization and navigation techniques have been used previously [18-25]. Graph visualization techniques address complexity and size of networks [26].

We demonstrate the usefulness of BISON through two examples within the *E. coli *network of transcriptional regulation. The first example uses FlhD/FlhC, a transcriptional regulator that was originally described as an activator of more than 50 flagellar genes [27] and later recognized as a global regulator of metabolism [28]. Expression of the *flhD *operon is a target point for many global regulators and global signals [29-32]. The portion of the *E. coli *transcriptional network that centers around FlhD/FlhC was summarized [33]. We will use this system to demonstrate how diverse data such as microarray data can be integrated with existing data and analyzed by BISON in the context of the entire regulatory network.

The second example focuses on ABC transporters, protein complexes that form continuous channels through both cellular membranes that are specific for certain substrates and require the hydrolysis of ATP to provide energy for the transport process (for a review, please, see [34]). Different regulators have been described for the regulation of the many ABC transporters [35]. To our knowledge, this study is the first attempt to summarize their regulations.

In summary, we take the study of biological networks beyond its traditional focus on network structure and move it towards a more function-oriented view that looks at meaningful patterns in a localized context and provides targeted information to biologists working on a limited number of genes.

## Results and discussion

**Figure 1:**
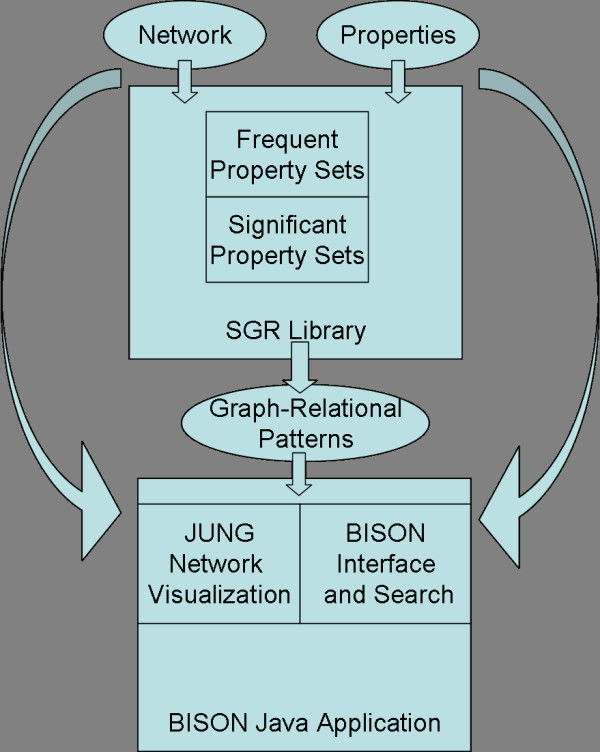
Schematic of the BISON data flow and design. Top part, underlying pattern mining engine; botton part, BISON interface.

This study presents an application [BISON; see Additional file 1] that combines our global pattern mining engine (an extension of [17]) with modern navigation and network visualization techniques [36,37]. Fig. 1 is a schematic of BISON. The underlying pattern mining engine is shown in the top portion of the Figure. It operates on the full network in a global fashion. The bottom part describes the BISON interface including a network visualization unit that uses graph navigation capability and navigation capability using a modern graphical user interface.

**Figure 2:**
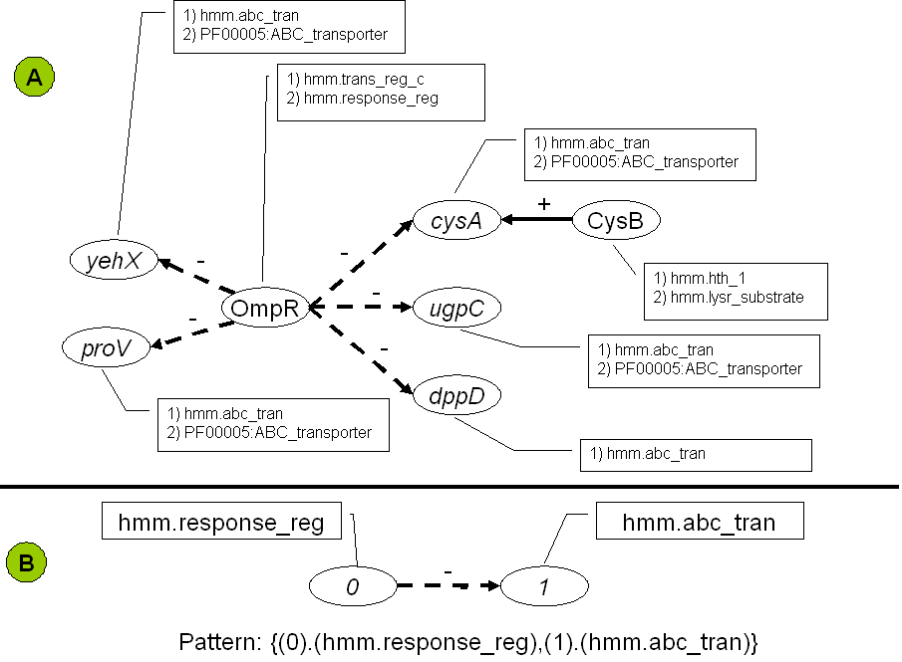
Concept of a graph-relational pattern. Panel A: Portion of a regulatory network with property data. Panel B, pattern from that network.

BISON connects regulatory network information with properties of the involved proteins. Properties can be any information that is associated with proteins, including experimental information and sequence information, such as annotations. The majority of the property data that are currently included in BISON are hidden Markov model domains (HMMs). Fig. 2A presents as small portion of such a regulatory network with the respective HMMs of the involved proteins.

We refer to the global patterns as graph-relational patterns. Fig. 2B illustrates the concept of a graph-relational pattern. Patterns are defined as frequently occurring combinations of properties in regulator proteins and the proteins that are encoded by their regulated genes. Patterns express structural attributes of the graph as well as specific properties of the relational records. Consequently, they form a bridge between regulation and function. Throughout this manuscript, properties for regulators are indicated (0) and properties for regulated proteins are indicated (1). The pattern in Fig. 2B, {(0).(hmm.response_reg),(1).(hmm.abc_tran)}, occurs at least five times within the entire network, a portion of which is displayed as Panel A.

It is important to note that BISON determines the frequency of occurrence of a certain pattern across the entire network and not only over the portion of the network that is displayed. Before a combination is considered a pattern, the combination has to occur at least a user specified number of times. The pattern repository currently provided with BISON is based on a cutoff of five times. It is also noteworthy that the pattern search is not limited to single properties on each site. All combinations of properties that satisfy the cutoff are being tested. While the current sub-graph type is set at single interactions (two proteins), the application also has the ability to find patterns that involve more than two proteins. Examples of these larger patterns are the hierarchical regulation of a final target gene, the simultaneous regulation of several genes with distinct properties, and co-regulation of a target gene by multiple regulators. Instructions on how to change these settings are provided with the User Manual that accompanies this manuscript [see Additional file 2].

**Figure 3:**
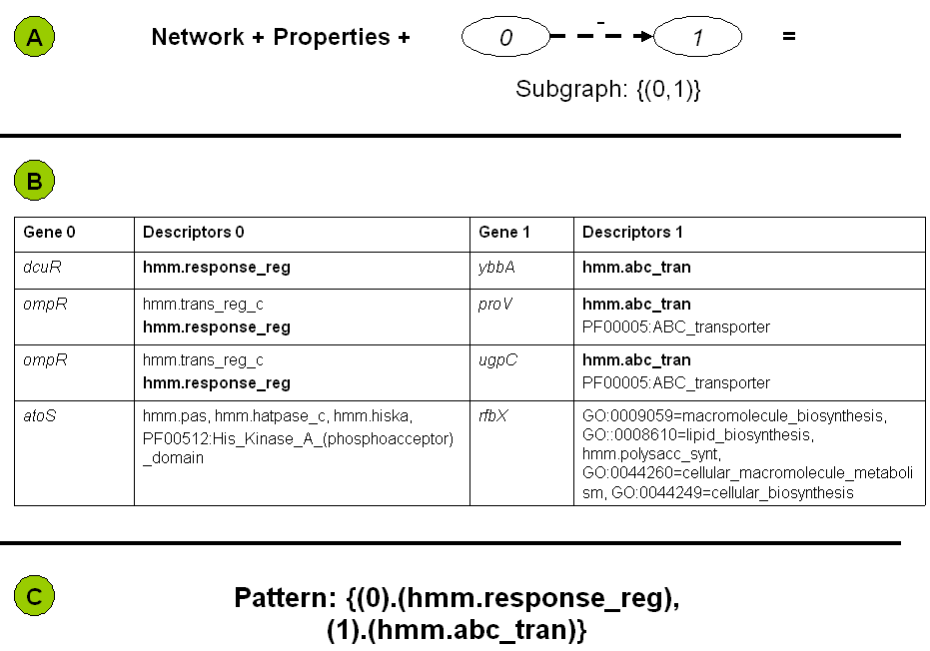
Details of a graph-relational pattern. Panel A: Network data, property data and the shape of the network define the combination of properties that can form a pattern. Panel B: Sampling of property combinations that have the potential to form patterns. Panel C: Pattern that was formed by the combinations (bold print) in Panel B.

Fig. 3 further details the concept of a graph-relational pattern. The combination of the network data, the property data, and the shape of the pattern (Panel A) will yield the combinations of genes that eventually form the pattern. Panel B includes a sampling of property combinations that have the potential of leading to patterns. The two columns labeled 'Descriptors' contain the property information. The two columns labeled 'Gene' contain the network information. Each gene encoding a regulator in the 'Gene 0' column is listed with the regulated gene in the 'Gene 1' column. The first line is a combination that contributes to the pattern. In this example, the pattern has a single property on each side. The second and third lines show how a pattern with multiple properties may be formed. The fourth line shows how gene ontology (GO) information can contribute to potential patterns. Of all the potential property combinations that are listed in Panel B, only the combination of the hmm.response_reg domain with the hmm.abc_tran domain (printed in bold) actually led to the pattern that is displayed in Panel C. The Pattern Information Page in BISON only lists such combinations of properties that actually form the pattern.

We now provide two examples of the type of biological questions that the pattern search engine of BISON can answer. Our examples will use the transcriptional network of the *Escherichia coli *K-12 strain. The first question asked is what functional categories of proteins are regulated by the global regulator FlhD/FlhC. This is an important question when analyzing microarray data, avoiding the publication of long lists of genes. For this purpose, the gene list of a previous microarray experiment [28] was integrated into BISON. Instructions on how users can integrate their own microarray data into BISON are included in the User Manual.

**Figure 4:**
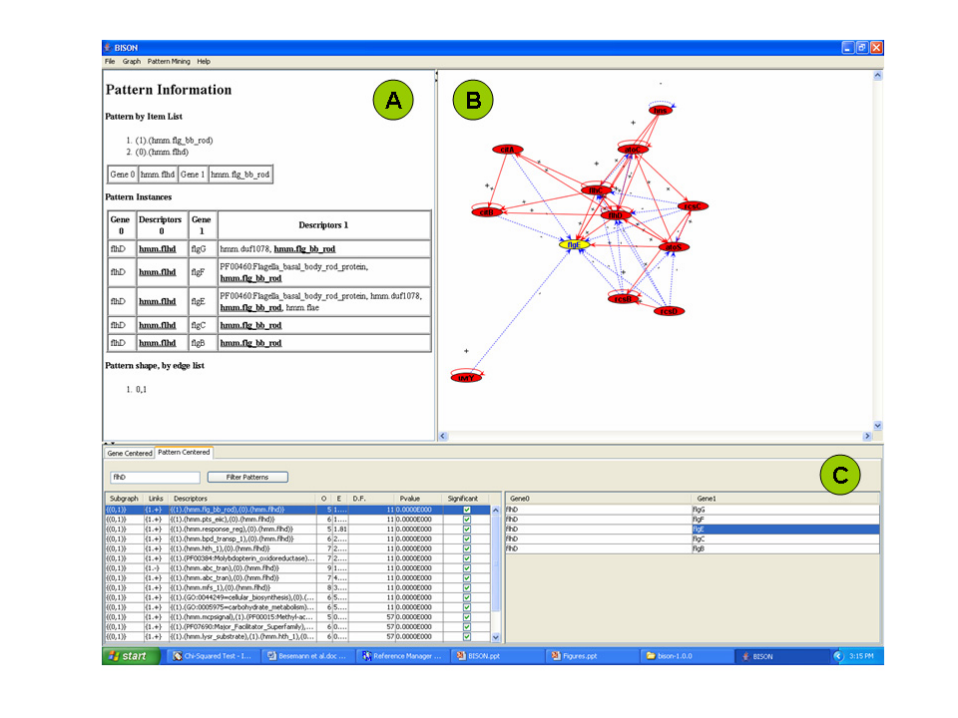
Pattern-centered screen shot of BISON. Panel A, Pattern Information page; Panel B, Network Visualization page; Panel C, Navigation page.

Using the pattern-centered analysis function of BISON (Fig. 4), we were able to determine all patterns that contain the hmm.flhd domain in the regulator protein (left portion of the navigation page), the genes that are associated with each pattern (right portion of the navigation page), and the full pattern information for each pattern (pattern information page).

Navigation page (Fig. 4C): The part of the 'Descriptors' column, designated (0), indicates properties found in the regulator, while the part, designated (1), indicates properties found in the regulated proteins. The 'Links' column indicates whether regulation is positive (+) or negative (-). Selecting a pattern on the left side will yield the associated genes on the right side. For example, the hmm.flhd domain frequently occurred in combination with the flg__bb_rod domain. The FlhD/FlhC regulated proteins that contain this domain are FlgB, FlgC, FlgE, FlgF, and FlgG.

**Figure 5:**
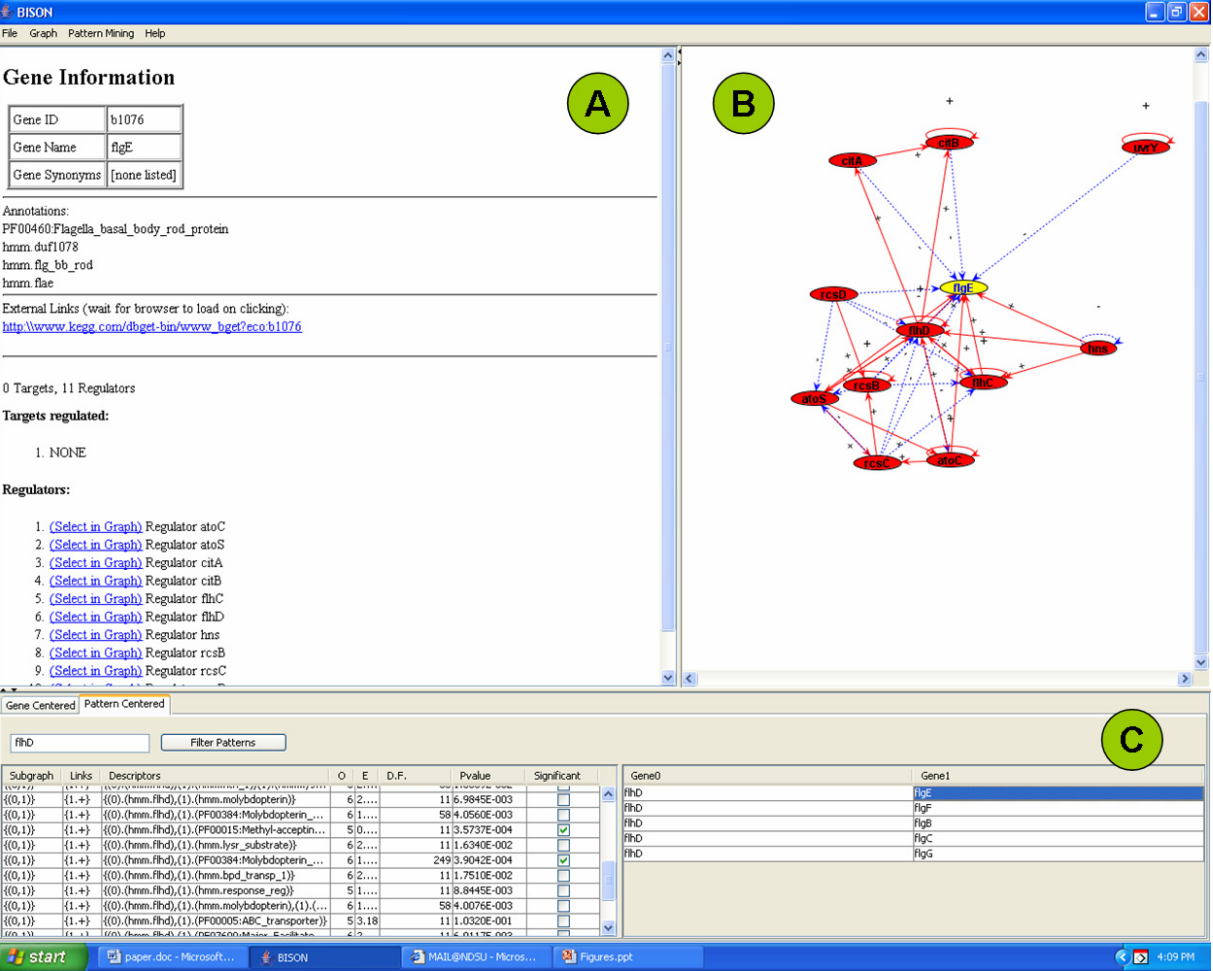
Gene-centered screen shot of BISON. Panel A, Gene Information page; Panel B, Network Visualization page; Panel C, Navigation page.

Network visualization page (Fig. 4B): Understanding of the patterns is supported by a visual display of the network environment. This display is limited to those proteins that have an immediate regulatory interaction with this gene to keep the computational and visual complexity under control. Selecting a gene on the right side of the navigation page will form the network around this gene in the network visualization page. This will also perform a switch from pattern-centered analysis to gene-centered analysis by converting the pattern information page into the gene information page (Fig. 5). This function is designed for users who want to further investigate properties of a gene or protein. A detailed list of genes and protein functions is now provided in Panel A. A link is provided to an external information source which is currently the Kyoto Encyclopedia of Genes and Genomes [38,39].

Pattern information page (Fig. 4A): This page contains a summary of all the information that is required for the pattern analysis. The columns are designated as described for Fig. 3B. The two 'Descriptors' columns contain the properties, the two 'Gene' columns the network information. In our example, FlhD/FlhC with the hmm.flhd domain regulates all the genes whose encoded proteins contain the hmm.flg_bb_rod domain (*flgC, flgD, flgE, flgF*, and *flgG*). This information can be obtained for all the patterns in the left portion of the navigation page.

Table 1 summarizes a selection of the results from the FlhD/FlhC analysis. Starting from Fig. 4, HMMs from the regulated proteins were taken from the 'Descriptors' column (labeled (1)) and regulated genes whose proteins contain this HMM were taken from the 'Gene 1' column. This was done for a selection of the patterns. For the sake of an easier discussion, proteins containing related HMMs are grouped into functional categories. For example, proteins that contain the hmm.flg_bb_rod domain and proteins that contain the hmm.mcpsignal domain are discussed together as flagellar proteins. Proteins that contain the hmm.pts_eiic domain and proteins that contain the hmm.abc_tran domain are discussed together as transport proteins. The terminal reductases of anaerobic metabolism contain up to three specific domains. These, too, are discussed together. Functional categories are indicated as bold printed subheadings in Table 1.

**Table 1:** Functional categories of proteins whose genes are regulated by FlhD/FlhC

**Regulator gene**^1^	**HMM of the regulator**^2^	**Regulated genes**^3^	**HMM of regulated proteins**^4^
**Flagellar proteins**			
*flhD*	flhd	*flgB, flgC, flgE, fleF, flgG*	flg_bb_rod
*flhD*	flhd	*tar, tsr, tap, trg, aer*	mcpsignal
			
**Anaerobic metabolism**			
*flhD*	flhd	*dmsA, ynfE, ynfF, napA, narG, narZ*	molydopterin_oxidoreductase, molydop_binding, molybdopterin
			
**Transport proteins**			
*flhD*	flhd	*nagE, frvB, treB, murP, mngA, chbC, malX, fruA, ptsG*	pts_eiic
*flhD*	flhd	*rbsA, oppD, nikD, fepC, proV, nikE, btuD, uup, metN, thiQ, phnK, msbA, fhuC, mglA, ybhF, dppD*	abc_trans
			
**Transcriptional regulators**			
*flhD*	flhd	*cynR, dsdC, xapR, tdcA, ydcI, ybbS, nhaR, argP, yeeV*	lysr_substrate, hth_1

^1^The gene 'flhD'.

^2^HMM for FlhD.

^3^Genes that are regulated by FlhD/FlhC are taken from the 'Gene 1' column in BISON that is associated with a selection of the property combinations in the 'Descriptors' column.

^4^HMMs for the regulated proteins are taken from the 'Descriptors' column in BISON.

Two functional categories were chosen for discussion as control groups of genes whose regulation by FlhD/FlhC was known. The remaining two functional categories were chosen as examples for functional categories that were newly recognized as regulated by FlhD/FlhC.

The two functional categories of proteins that were known to be regulated by FlhD/FlhC are flagellar proteins and anaerobic metabolism [28]. The group of flagella proteins that are covered with this analysis, includes the above mentioned flagella basal body rod proteins (hmm.flg_bb_rod) and the methyl-accepting chemotaxis proteins (hmm.mcpsignal). Since FlhD/FlhC was initially described as a transcriptional regulator of all flagellar genes [27], this confirms previous results and will serve as our primary positive control. Please, note that FlhD/FlhC regulates more flagellar genes than just the ten genes that are listed in Table 1. A complete list of FlhD/FlhC regulates genes can be obtained with the gene-centered analysis (gene information page). Pattern-centered analysis can lead to further hypotheses on domains that co-regulated genes share. Lowering the cutoff from five to three (number of combinations that are considered a pattern) yields the hmm.duf domain within FlgK, FlgG, and FlgE. This is so far a domain of unknown function. The observation that it is common among FlhD-regulated proteins may assist in determining its associated function.

As a secondary positive control, we confirmed the effect of FlhD/FlhC upon enzymes of anaerobic metabolism. As published before [28], several proteins that contain the HMMs characteristic for terminal reductases were regulated by FlhD/FlhC. Interestingly, BISON identified two proteins of this category (YnfE, YnfF: [40]) as regulated by FlhD/FlhC that were missed by the previous analysis [28].

In addition to the controls mentioned above, we were able to establish new information using BISON. Two functional categories of genes regulated by the regulator of interest, FlhD/FlhC, were better defined or newly established, transporters and transcriptional regulators. While it was previously recognized [28] that FlhD/FlhC regulated many genes encoding transporters, no attempt had been undertaken to classify these transporters. Our current analysis revealed two HMMs within the functional category of transport proteins that were regulated by FlhD/FlhC. These are pts_eiic (phosphotransferases) and abc_tran (ABC transporters). Each HMM is found in many proteins whose genes are regulated by FlhD/FlhC (Table 1). The HMM lysr_substrate in combination with hth_1 (helix-turn-helix) is an indication for DNA binding and transcriptional regulation. Nine proteins contain these two HMMs, all of these are regulated by FlhD/FlhC. This functional group of transcriptional regulators is a very interesting new finding, supporting our idea of FlhD/FlhC being part of a larger network of transcriptional regulation [33].

In summary, BISON identified two functional categories that were previously described as regulated by FlhD/FlhC (flagellar proteins and anaerobic metabolism), further detailed the category of transport proteins, and identified a new functional category, transcriptional regulators. In addition, new proteins were recognized as regulated by FlhD/FlhC in the category that relates to anaerobic respiration. We interpret this as an indication that pattern mining is a useful tool for the analysis of complex microarray and network data.

The second biological question that was asked was what functional categories of proteins (indicated by their HMM) are involved in the regulation of ABC transporter genes. A search was performed for patterns containing 'ABC'. After selecting a pattern involving 'ABC', the hmm.abc_tran domain appears in the 'Descriptor' column labeled (1). HMMs of proteins that regulate ABC transporter genes were taken from the 'Descriptor' column, labeled (0). The genes encoding these regulators were taken from the 'Gene 0' column. The specific ABC transporter genes that are regulated by this regulator were taken from the 'Gene 1' column. These regulations are summarized in Table 2 and correspond to the Pattern Information page.

**Table 2:** Regulators that affect the expression levels of ABC transporter genes

**Regulator gene**^1^	**HMM of the regulator**^2^	**Regulated genes**^3^	**HMM of regulated proteins**^4^
**Two-component systems**			
			
*torS*	response_reg	*fepC*	abc_tran
*phoB*	response_reg	*ugpC, pstB, phnL, phnK, phnC*	abc_tran
*narL*	response_reg	*cydC, cydD, ccmA*	abc_tran
*narP*	response_reg	*ccmA*	abc_tran
*rcsB*	response_reg	*tauB, fepC, ycjV, nikD, nikE, malK*	abc_tran
*torR*	hiska	*fepC*	abc_tran
*rcsC*	hiska, response_reg	*tauB, fepC, ycjV, nikD, nikE, malK*	abc_tran
*ompR*	response_reg	*yehX, cysA, proV, ugpC, dppD*	abc_tran
*envZ*	hiska	*yehX, cysA, proV, ugpC, dppD*	abc_tran
*glnG*	hth_8	*glnQ, potG, dppF, dppD, hisP, yhdZ*	abc_tran
			
**DNA binding proteins**			
			
*modE*	hth_1	*modC, ccmA*	abc_tran
*cysB*	hth_1	*tauB, cysA*	abc_tran
*cbl*	hth_1	*tauB*	abc_tran
*oxyR*	hth_1	*sufC*	abc_tran

^1^Genes encoding regulators that affect the expression levels of ABC transporter genes are taken from the 'Gene 0' column on the navigation page.

^2^HMMs for the regulators are taken from the 'Descriptors' column on the navigation page. HMMs are indicative of the functional categories of regulators (bold subheadings) that affect the expression levels of ABC transporters.

^3^Genes encoding ABC transporters that are affected by the regulators in column 2 are taken from the 'Gene 1' column on the navigation page.

^4^HMMs for the regulated proteins are taken from the 'Descriptors' column on the navigation page. HMMs are indicative of ABC transporters.

Proteins regulating ABC transporters were grouped into functional categories again, based upon their properties (HMM). Two-component systems each consist of a histidine kinase (hmm.hiska) and a response regulator (hmm.response_reg) (for a review, please, see [41]). RcsCDB is a rare case of a three-component system, where the first component (RcsC) contains both functional domains [42]. In the cases of RcsCDB, EnvZ/OmpR, and TorS/TorR, histidine kinases and response regulators have been identified as regulators of ABC transporter genes (Table 2). In the cases of PhoR/PhoB, NarX/NarL, NarQ/NarP, and NtrB/NtrC (synonym GlnG that was identified as a regulator of ABC transporter genes by its hth_8 domain), only the response regulator was found to regulate ABC transporter genes. Overall, the contribution of two-component systems to the regulation of ABC transporters seems to be large. Considering the small degree of overlap between the regulated genes, it seems like many two-component systems are specific for a certain set of ABC transporter genes.

An example of how BISON can be used to create hypotheses that can be further examined experimentally is given as an extension of the above study and involves the second functional categories of proteins that regulate ABC transporter genes, DNA binding proteins: ModE (hmm.hth_1) is a known repressor of the *modA *operon that encodes a molybdate specific transporter [43] and the *ccmA *operon that encodes a haem transport system [44]. While early studies with the *ccmA *operon showed that transcription was induced during anaerobic growth, regulation by known regulators of anaerobic respiration (FNR, ArcB/ArcA) could not be detected [44]. With this study, we found a regulation of *ccmA *by NarX/NarL and NarQ/NarP (Table 2). Both these two-component systems are global regulators during anaerobic growth in the presence of nitrate [45]. This leads to the hypothesis that regulation of *ccmA *by these systems might explain the regulation by anaerobiosis. Further experimentation might confirm this hypothesis.

**Figure 6:**
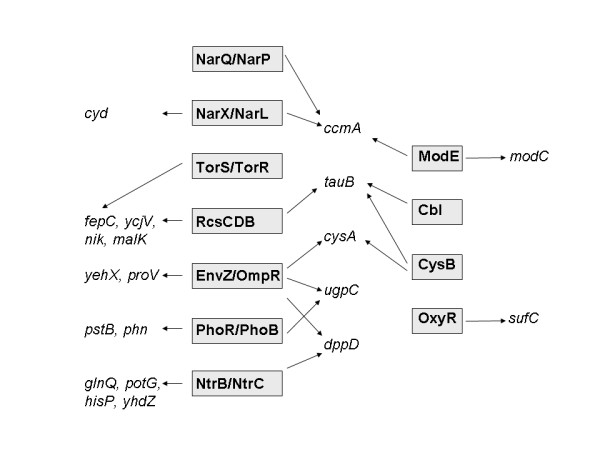
Schematic of the regulation of ABC transporters. Regulators are boxed, regulated ABC transporter genes are in italics.

In summary, regulation of ABC transporters seems to be as global as any regulation of metabolism. Interactions between regulators and their target genes are summarized in Fig. 6.

## Conclusion

We have developed a tool for the analysis of networks and global patterns. BISON is global in its design, yet allows the biology user to ask specific questions. We provide two examples for the kind of questions that can be answered with BISON and examine results from a microarray experiment in the context of the existing regulatory network. We also provide an example of how BISON can be used to create hypotheses. BISON provides a function oriented analysis for complex data. It bridges a gap between studies of single interactions and global pattern mining techniques. For this reason, we call our analysis 'semi-global'. Access to BISON is provided through BioMed Central [Additional file 1] and the NDSU Computer Science website [46].

## Methods

### Components of BISON

BISON is a tool designed to support exploration and graphical visualization of network patterns. It was developed in the Java 5.0 programming language. The schematic in Fig. 1 illustrates the structure of BISON. BISON takes raw network and property input as well as results provided by our own external Perl library for frequent (graph-relational) pattern mining (SGR Perl Library). We consider differential patterns that express differences in the properties of connected nodes as discussed [17]. In addition, patterns that represent similarity across edges are also discovered. Similarity and dissimilarity patterns are treated separately. This allows us to maintain the expression power of patterns we developed earlier [17] and yet to provide a larger set of patterns to the user. BISON is divided into a graphical visualization part and a text database interface for analysis and search. The network visualization component makes use of the Java Universal Network/Graph (JUNG) Framework [37]. The source code for BISON, documentation, and the pattern library are contained in the supplemental files.

### Sources for network data

Network data were obtained from public databases, as well as experiments of our own and other researchers. A summary is given in Table 3. Four sources are described as follows: RegulonDB [2,47] provides 2,537 interactions defined by 142 regulators and 1,059 targets. Microarray data for all of the *E. coli *two-component systems [48] contain 1,028 interactions between 40 regulators and 372 targets. A previous compilation of data [33] contains 1,969 interactions over 26 regulators and 856 targets. Finally, a previous microarray experiment from our own laboratory [28] contributed 886 interactions over 2 regulators and 444 targets. The total number of interactions is 6,227 between 186 regulators and 1,934 targets. These sources were formatted for use in BISON. Since we wanted to link network data to node property information, we resolved all gene names to a common identifier (Blattner IDs [49]). Gene names that could not be linked to Blattner IDs were excluded from the network since they could not contribute to useful patterns.

**Table 3:** Network data

**Source**	**Interactions**	**Regulators**	**Regulated genes**	**Reference**	**File name**
RegulonDB	2,537	142	1,059	[2,47]	regulon.net
Two-comp.	1,028	40	372	[48]	2component.net
Compilation	1,969	26	856	[33]	pruess.net
FlhD/FlhC	896	2	444	[28]	flhD_microarray.net
					
**Total**	**6,227**	**186**	**1,934**		

### Sources for property data

Property data were obtained from public sources, supplemented by our own set of properties. A summary is given in Table 4. The initial property data were obtained from the *E. coli *genome project [49,50] that includes 4,285 proteins and contains Gene Ontology (GO) classifications. The project yielded a total of 62 GO annotations (prefix "GO" in BISON). To extend these data, we searched the protein sequences for protein family domains. First, we gathered default data from the Pfam site [51,52] from which we extracted 1,032 annotations covering 2,271 proteins (prefix "PF" in BISON). In addition, we used the HMMER profile hidden Markov model software [53,54] that is used by Pfam to identify potential domain annotations using different criteria. Sequences from the *E. coli *Genome Project [49,50] were tested with the Pfam A domain models. Domains were detected at a cut-off e-value of 1e-10. From this software setup, we extracted 1,747 annotations covering 3,124 proteins (prefix "hmm"). Note that some proteins received multiple HMM annotations and some annotations overlap with the default Pfam annotations.

**Table 4:** Annotation data

**Source**	**Annotations**	**Proteins**	**Reference**	**Designation**
*E. coli *Genome Project	62	106	[49,50]	GO
Pfam	1,032	2,271	[51,52]	PF
HMMER	1,747	3,124	[53,54]	HMM
				
**Total**	**2,841**	**3,495**		

### Data input files

All input data are collected in a single data directory (default_data). The directory includes a configuration file (bison.config) read by BISON that specifies names of data files. Files required by BISON: An entity file (ecoli_entity.txt) lists the nodes of the network and the set of properties available for each node. Specific node IDs (Blattner IDs) are gathered from this file. An alias file (ecoli_alias.txt) specifies the default gene names for the nodes. A synonym file (ecoli_syn.txt) lists additional names for the nodes. Finally, a pattern file (patterns.out) stores the patterns of entities and annotations discovered in the network. This file is generated by the pattern mining engine. BISON can accept results from other pattern generation libraries, provided the format requirements of the pattern output file are satisfied. The configuration file also contains a list of external web links that are used in combination with protein IDs to construct hyperlinks in the object information page.

Four network files (*.net) contain data from the four different sources we used (Table 3). Additional network files can be added to allow the user to integrate their own data and understand them in the context of the existing network. A line will have to be added in the bison.config file that lists the name of the new data file. Similarly, protein property information can be supplemented. If additional data are to be included in the pattern discovery, then the pattern mining script has to be re-run with the new data. This will result in a new pattern file (see above). Instructions for the addition of user-specific files are contained in the User Manual.

### JUNG Java extensions

We extended the JUNG Java library [37] as a basis for the graph visualization component. By default, the Fruchterman-Reingold node layout [36] is used for graph visualization if the displayed portion of the graph is smaller than 100 nodes, and a circle layout is used otherwise. BISON also resizes nodes to fit into the visualization space. Since this can cause problems with reading labels on the graph, text information is also provided in the application.

### Pattern mining

Graph-Relational pattern analysis (Fig. 2, top portion, indicated as SGR) is implemented as a Java library within BISON. A summary of the mining process is outlined below:

1. Input network data and property data

2. Join the input data according to the requested pattern shape

3. Find frequent patterns in the joined table of data

4. Compute statistical significance measure

5. Output patterns for use in BISON

Step 1: Input files for the pattern mining contain the network data and the property data. The goal of pattern mining is to characterize interaction structures given as pattern shapes or subgraphs and the properties associated with the nodes involved in those structures. Pattern shapes can be pairs of regulators and regulated genes, or regulatory structures of three or more proteins.

Steps 2 and 3: The algorithm is an extension of our previous work [17]. In addition to analyzing differences between nodes, this algorithm allows the study of similarities. Patterns are separated into groups that represent combinations of similar and dissimilar annotations between different nodes in the network structure.

Step 4: The *p*-value for frequent patterns from step 3 is calculated, using a Chi-Squared test based on the contingency table of all items in the pattern. The objective of the test is to determine whether the pattern could have occurred randomly. We consider independence of all items in the pattern as null hypothesis. Note that both absence and presence of properties defines a pattern (see [17] for a discussion). That means that for a pattern (0).A (1).B in a 1-edge shape a four-dimensional contingency table is constructed based on variables 0.A, 1.A, 0.B, 1.B. Note also that a pattern may appear as significant because of co-occurrence of items within individual proteins. A *p*-value of 0.001 was chosen as cutoff. Based on this cutoff, all patterns that were discussed in the manuscript are significant. We are currently working on algorithms to specifically determine the significance of patterns with respect to the regulatory interactions and take the sparseness of the data into account.

Step 5: Writes the results to an output file (patterns.out) that is compatible with BISON.

Publication of the BISON application as part of an Open Access journal and making the code available in an Open Source format helps to facilitate the accessibility of the techniques. We will deliver updates and improvements as the BISON application progresses. We appreciate citation of this publication for users who want to include results obtained with BISON in their manuscripts.

## Abbreviations

BISON, Bio-Interface for the Semi-global analysis Of Network patterns; HMM, Hidden Markov Model; JUNG, Java Universal Network/Graph Framework; KEGG, Kyoto Encyclopedia of Genes and Genomes; SGR, Significant Graph-Relational pattern mining library.

## Competing interests

The author(s) declare that they have no competing interests.

## Authors' contributions

AD is an assistant professor in the Department of Computer Sciences and CB is a graduate student in her research group. CB and AD developed BISON and wrote the computer science part of the manuscript.

BMP is an Assistant Professor in the Department of Veterinary and Microbiological Sciences and NC is an undergraduate student in her research group. NC and BMP performed the biological analysis of the data. NC was involved in the data acquisition and BMP wrote the biology part of the manuscript. All authors read and approved the final manuscript.

## Supplementary Material

Additional File 1 The BISON package contains the BISON application, example data from the paper, and the source code for BISON and the SGR pattern mining Perl library. The contents of the archive are further explained within the README.TXT file. Click here for file

Additional File 2 The User Manual contains instructions on how to use BISON, change pattern mining settings, and import the user's own data. Click here for file
