# Supplementary material for: BISON: bio-interface for the semi-global analysis of network patterns
Source: Source Code Biol Med. 2006 Nov 29;1:8. doi: 10.1186/1751-0473-1-8 (PMC1698476; doi:10.1186/1751-0473-1-8)
Supplement: Additional File 2 — The User Manual contains instructions on how to use BISON, change pattern mining settings, and import the user's own data. [file 1751-0473-1-8-S2.pdf]

BISON: Bio-Interface for the Semi-global analysis Of Network Patterns

User Manual

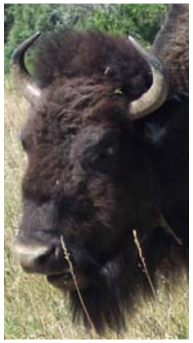

Christopher Besemann, Anne Denton, Nathan J. Carr, Birgit M. Prüß
North Dakota State University

BISON 1.0

- BISON is a software for the analysis of transcriptional networks of regulation. It combines a pattern mining engine with modern navigation and network visualization techniques.
- The current default directory of data files contains data from *Escherichia coli* K-12.
- BISON enables the user to load their own microarray data into the default directory to be analyzed in the context of the network.
- Data for other species can be loaded into BISON, constructing a new file directory.

Contacts

Christopher Besemann and Anne Denton
Department of Computer Sciences
IACC 258
1301 12th Ave N
North Dakota State University
Fargo ND 58105
Phone (701) 231-6748
E-mail: Anne.Denton@ndsu.edu

Nathan J. Carr and Birgit M. Prüß
Department of Veterinary and Microbiological Sciences
Van Es Hall 108
1523 Centennial Blvd.
North Dakota State University
Fargo ND 58105
Phone (701) 231-7848
E-mail: Birgit.Pruess@ndsu.edu

BISON 1.0

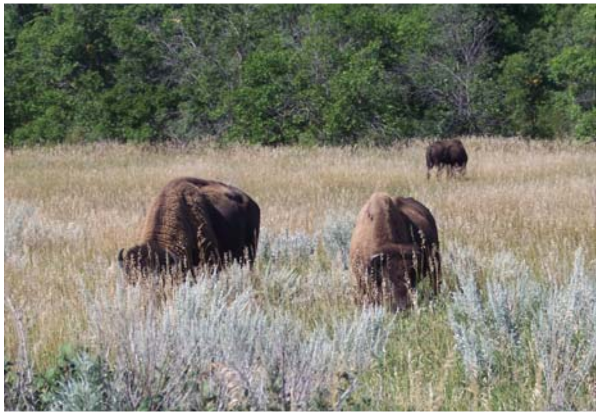

Software description

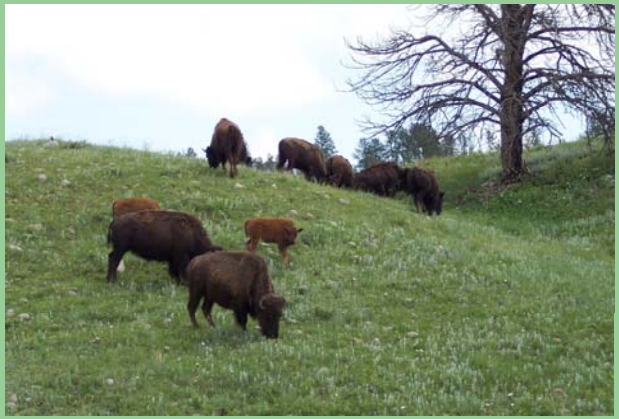

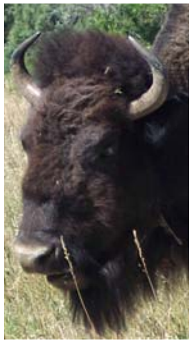

System Requirements

- Minimum system requirements:
  - System: 2 GHz, 1 GB of RAM
  - Operating system: Windows XP
- BISON might work with slower processors and other operating systems

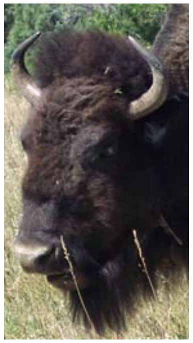

Loading BISON

- Installation is not necessary for BISON
- Install Java 5.0 (or higher) on your computer
  - http://www.java.com
  - Hit ‘Download’ now and then ‘Begin Download’
- Download the bison1.zip file from:
  - Source Code for Biology and Medicine
  - http://denton.cs.ndsu.nodak.edu/bison/
- Extract the files into an uncompressed directory so that the original folders are preserved
  - Select bison1.zip and open
  - Right mouse click bison1 and select ‘extract’
  - Choose ‘All files’, hit ‘extract’
  - A new folder will appear on your desktop, named bison1

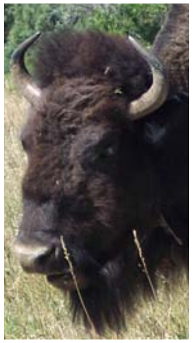

Configuration files

- Within bison1, the single data directory (default_data) contains two configuration files:

- Edge Color file (edgeColors.txt)

LABEL	RED_VALUE	GREEN_VALUE	BLUE_VALUE	ALPHA	DOTTED/SOLID
+	1f	0f	0f	1f	SOLID
-	0f	0f	1f	1f	DOTTED
+-	1f	1f	0f	1f	SOLID

- Configuration file (bison.config)

```
# "E.coli" from RegulonDB and Dr. Pruess data with annotations from Pfam and Wisconsin  GENE ID
ENTITYFILE      ecoli_entity.txt
ALIASFILE       ecoli_alias.txt
SYNONYMFILE  ecoli_syn.txt
PATTERNFILE   patterns.out
NETWORKFILE  flhD_microarray.net          edgeColors.txt
NETWORKFILE  regulon.net                  edgeColors.txt
NETWORKFILE  pruess.net                   edgeColors.txt
NETWORKFILE  2component.net               edgeColors.txt
LINK  http:\\www.kegg.com/dbget-bin/www_bget?eco:          ID
```

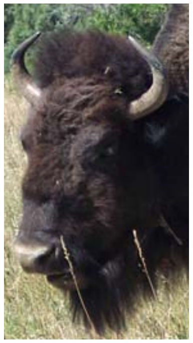

Data input files

- The single data directory (default_data) contains five data input files:
  - Entity file (ecoli_entity.txt) lists nodes of the network and the set of properties for each node
  - Alias file (ecoli_alias.txt) specifies the default gene names for the nodes
  - Synonym file (ecoli_syn.txt) lists additional names for the nodes
  - Pattern file (patterns.out) stores the patterns of entities and properties discovered in the network
  - Network files (*.net): list the edges (interactions) in the network.

Please, note that the patterns.out file is generated by BISON

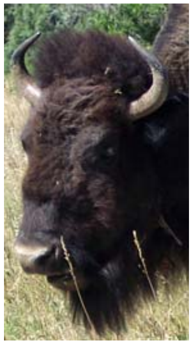

Data, regulation

Interaction data were integrated into the network files from the following sources:

Source	Interactions	Regulators	Regulated genes	Reference	File name
RegulonDB	2,537	142	1,059	Salgado *et al*., 2006	regulon.net
Two-comp.	1,028	40	372	Oshima *et al*., 2002	2component.net
Compilation	1,969	26	856	Prüß *et al*., 2006	pruess.net
FlhD/FlhC	896	2	444	Prüß *et al*., 2003	flhD_microarray.net
**Total**	**6,227**	**186**	**1,934**		

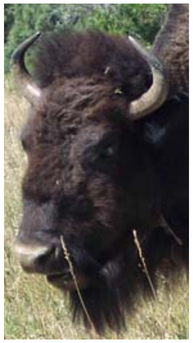

Data, properties (annotation)

Property data were integrated into the entity file from the following sources:

Source	Annotations	Proteins	Reference	Designation
E. coli Genome Project	62	106	http://www.genome.wisc.edu/	GO
Pfam	1,032	2,271	http://www.sanger.ac.uk/Software/Pfam/	PF
HMMER	1,747	3,124	http://hmmer.wustl.edu/	HMM
**Total**	**2,841**	**3,495**		

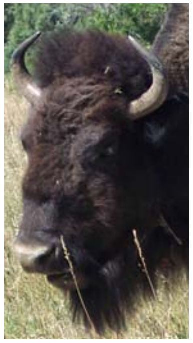

Opening BISON

- Exit ‘Default.data’ directory
- Click ‘Bison.exe’
- Click ‘File’, select ‘Load File Directory’ and select ‘Default_Data’

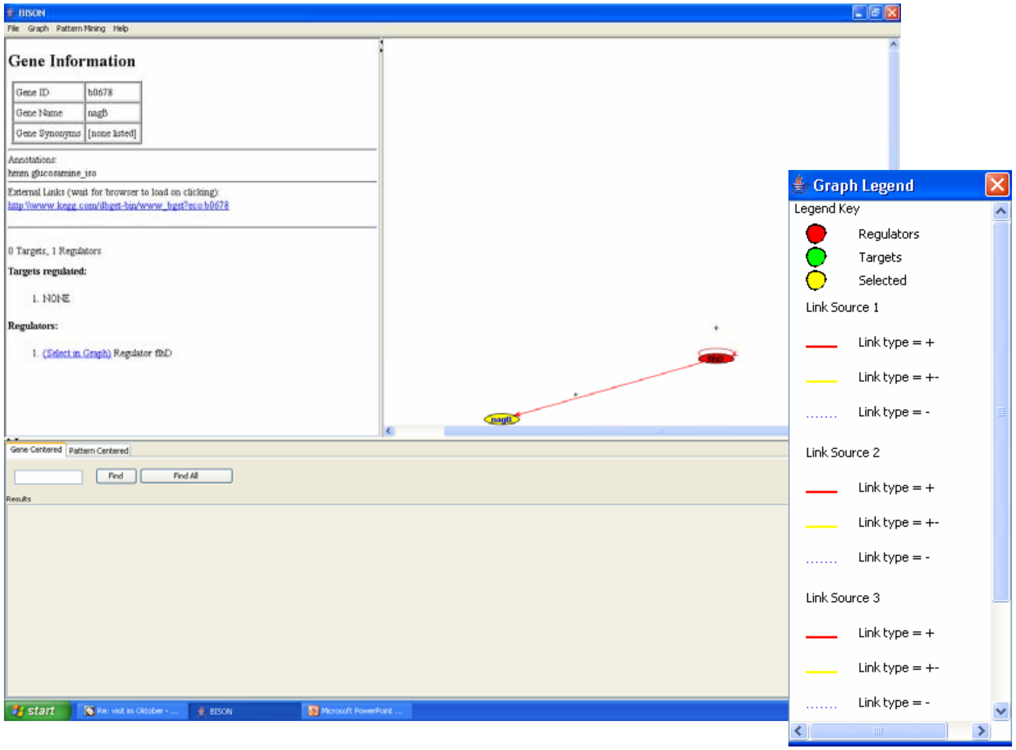

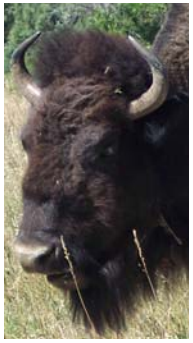

The BISON interface

- Top left page: object information page
- Top right page: network visualization page
- Bottom page: navigation page

- Legend: click upper left X to close, go to ‘Graph’ to re-open

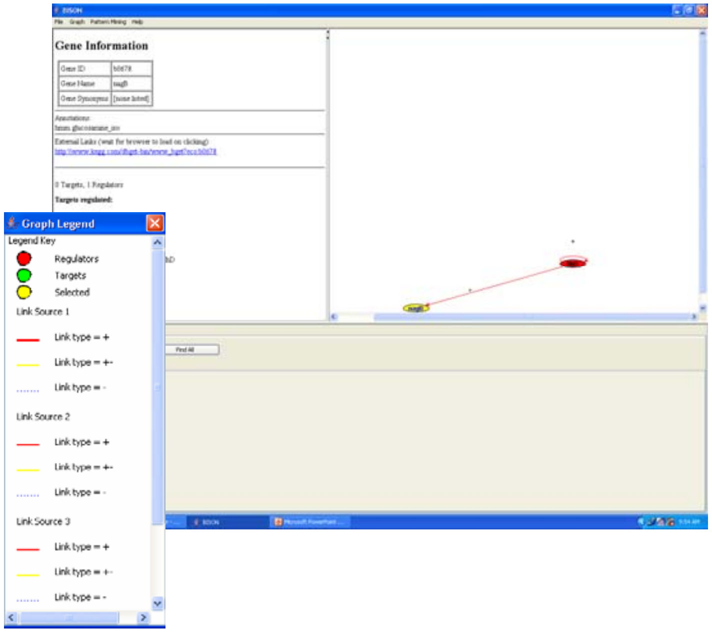

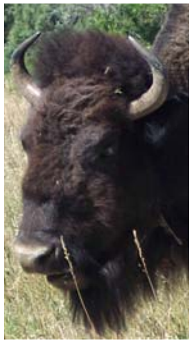

The navigation bar

- File: lets you select your data directory or exit
- Graph: lets you select the legend, choose a layout, select edge filters, zoom in and out
- Pattern mining: lets you select the pattern mining option
- Help: contains the help function

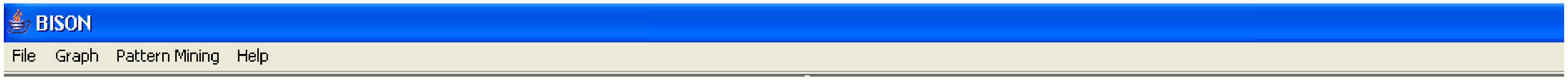

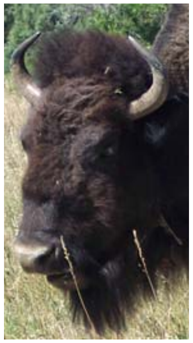

Legend

- Opens from the ‘Graph’ tab
- The legend explains the nodes and edges:
  - Red: regulator genes
  - Green: target genes
  - Yellow: selected gene
- The four link sources resemble the four *.net files (see pages 8 + 9)

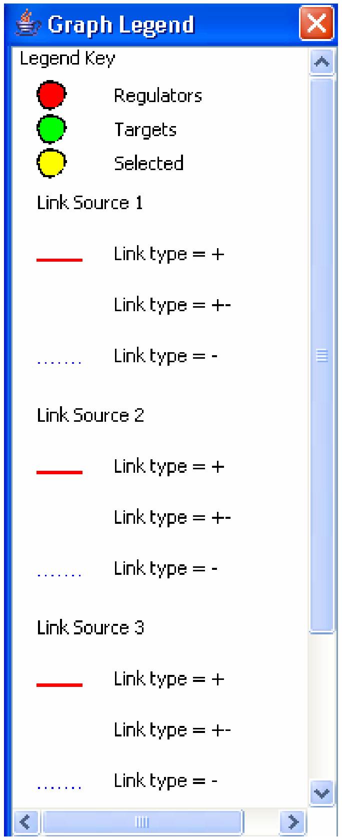

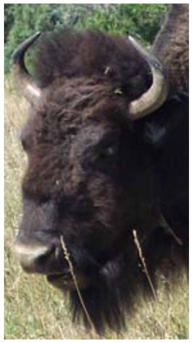

Layout options

- From the ‘Graph’ tab
- You get to select either the Fruchterman-Reingold (FR) layout or the circle layout

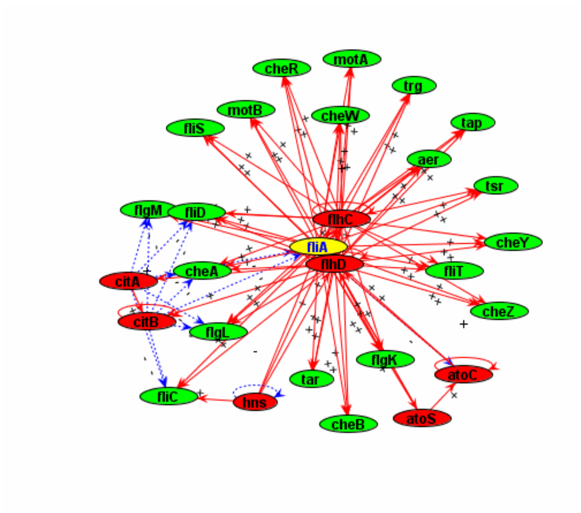

FR layout

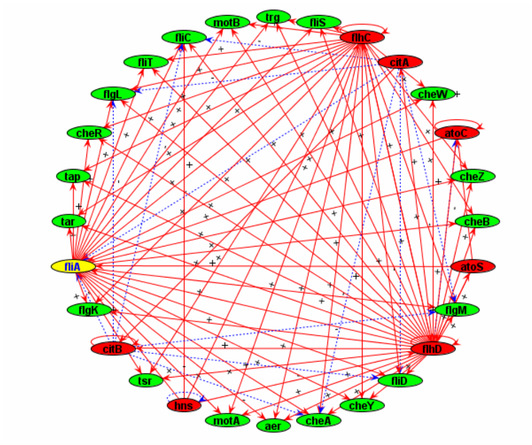

Circle layout

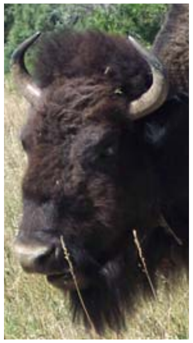

Edge filters

- From the ‘Graph’ tab
- You get to select either context edges or incident edges. Context edges show the connections between all nodes on the graph. Incident edges show only edges leaving or entering the selected node.

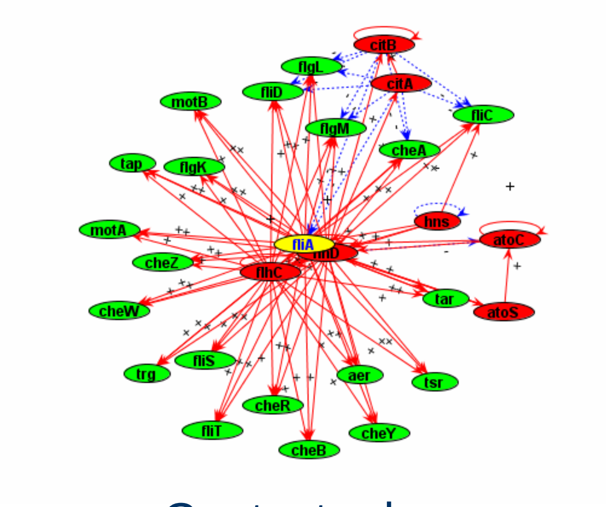

Context edges

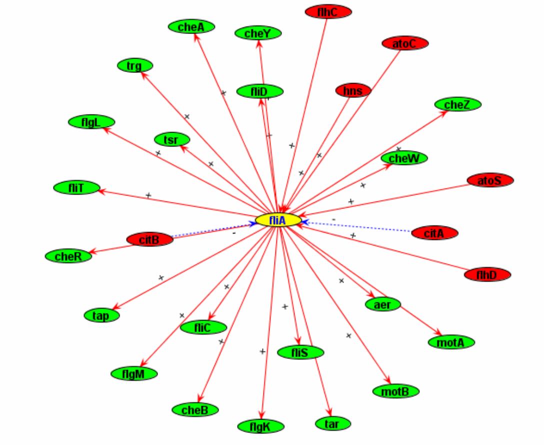

Incident edges

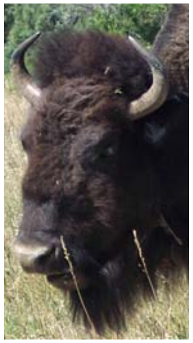

Zoom

- Select ‘Zoom’ from the ‘Graph’ tab
- On the bottom panel of the Satellite Viewer, you can choose between ‘zoom in’ and ‘zoom out’
- The white window lets you navigate within ‘Satellite Viewer’ to select your view area
- This function is particularly useful for networks that contain many nodes. Please note, that it will be slower then

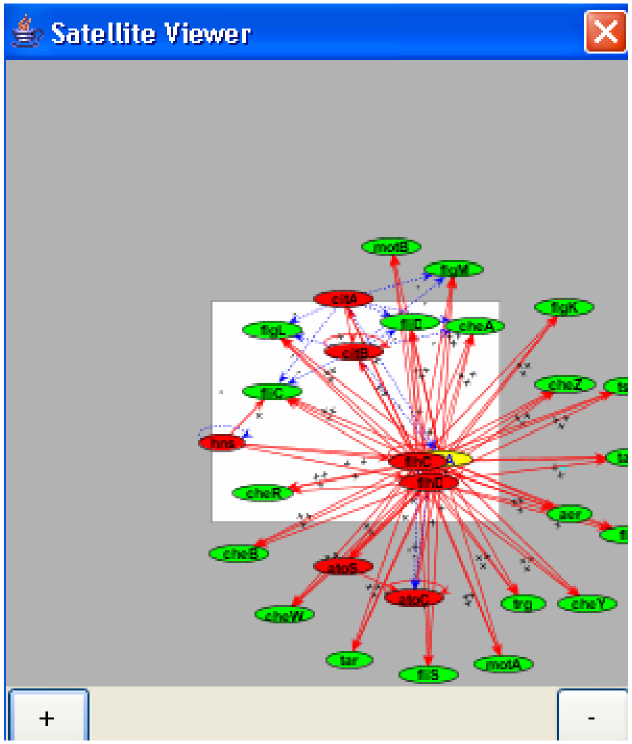

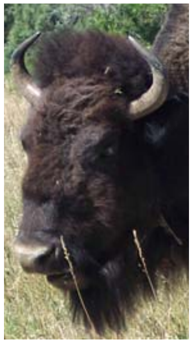

Pattern mining

- Copy the patterns.out file to a backup file (see page 8)
- Select ‘Pattern mining’ from the ‘Graph’ menu
  - Choose minimum pattern occurrence (this is your cutoff for meaningfulness of patterns)
  - Choose sub-graph file (1-edge indicates two proteins, 2-edge indicates three proteins)
  - Selecting ‘Compute Significance’ will run a Chi-squared test on the patterns (this takes about an hour)
  - Hit ‘Compute’
- BISON will now run the pattern library and calculate a new patterns.out file
- You will have to reload the data

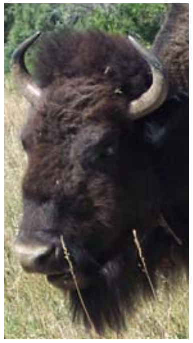

Resizing the pages

- Left click and drag the border lines of the network visualization page

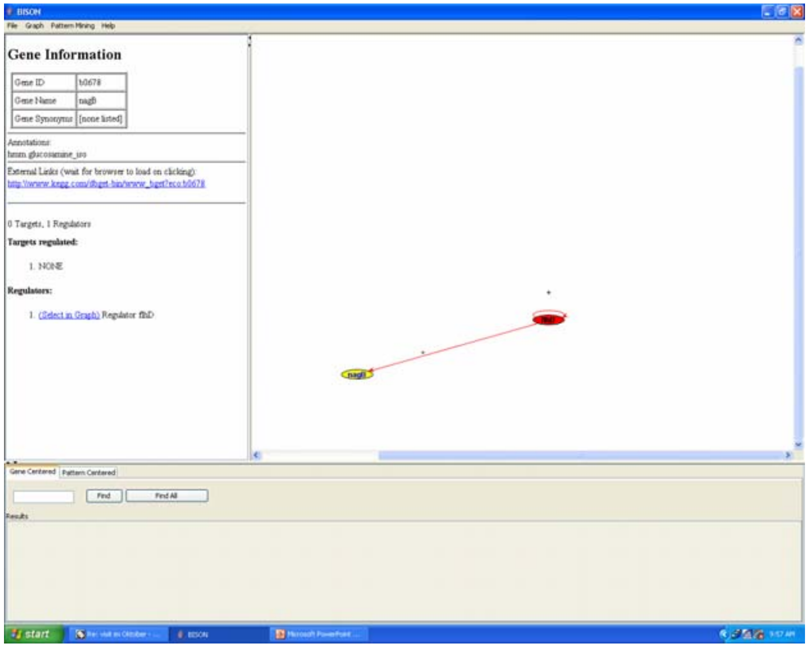

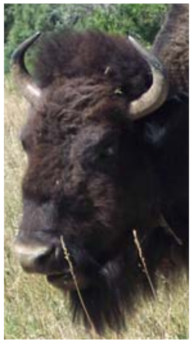

Gene-centered analysis

- Select the ‘Genes’ tab in the navigation page
- Use the ‘Find’ option to find your gene of interest. Click ‘Select in graph’

- Top left screen: the object information page will be the gene information page
- Top right screen: network visualization page
- Bottom screen: navigation page

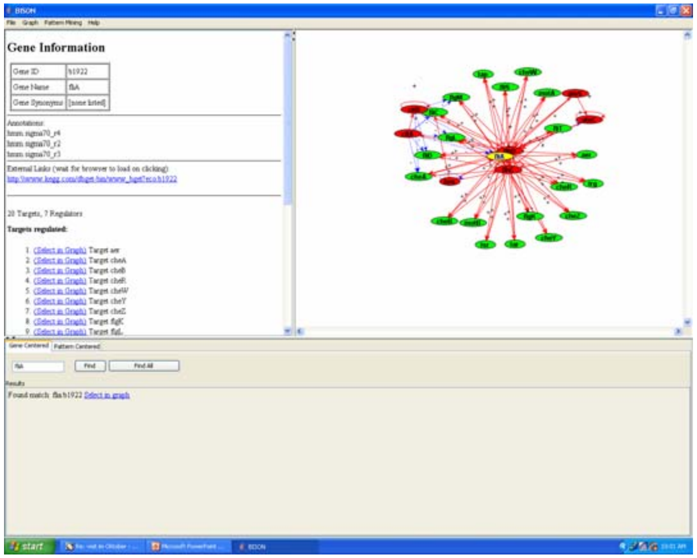

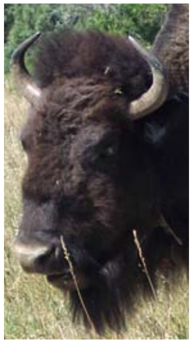

Navigation page

- The two tabs are for gene-centered and pattern-centered analysis

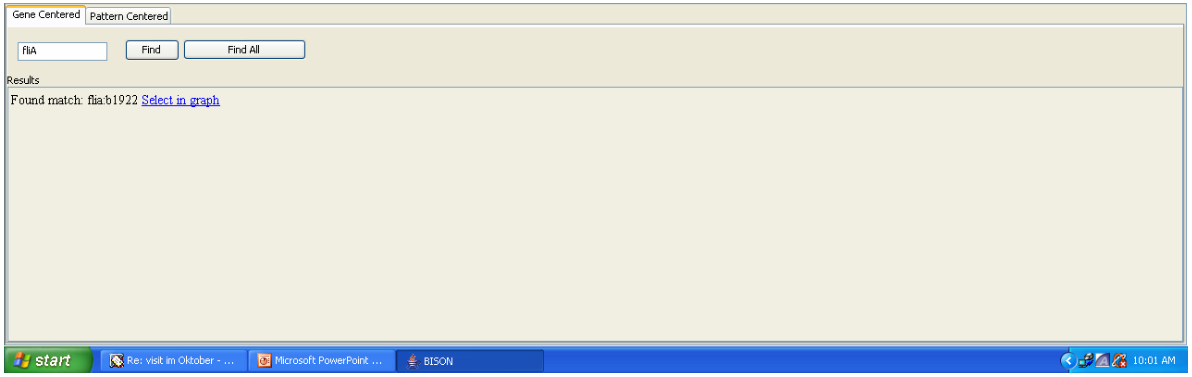

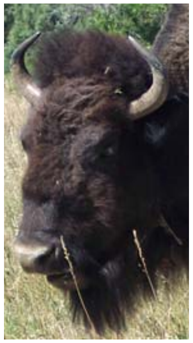

Network visualization page

- Yellow node in center: selected gene
- Red nodes: genes that serve as regulators
- Green nodes: genes that serve as regulated genes

- Red solid arrows: positive regulation
- Blue dotted arrows: negative regulation

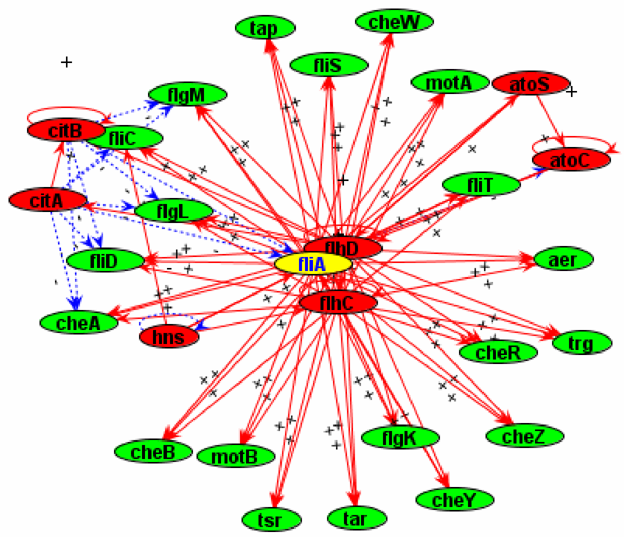

Please note: for regulators that affect the expression of a large number of genes, you will need to use the gene lists in the gene information page for your analysis (see next page). Also, use the 'Zoom' function.

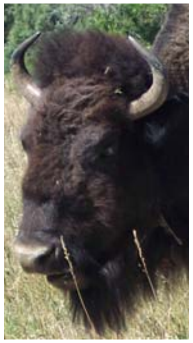

Gene information page

- Indicates properties that are associated with the proteins that are encoded by the selected gene
- Click the link for more information about this gene. You will connect to an external data source
- Lists all the target genes and the regulator genes of the selected gene. Selecting the link for any one of these genes will re-form the network visualization page around this gene

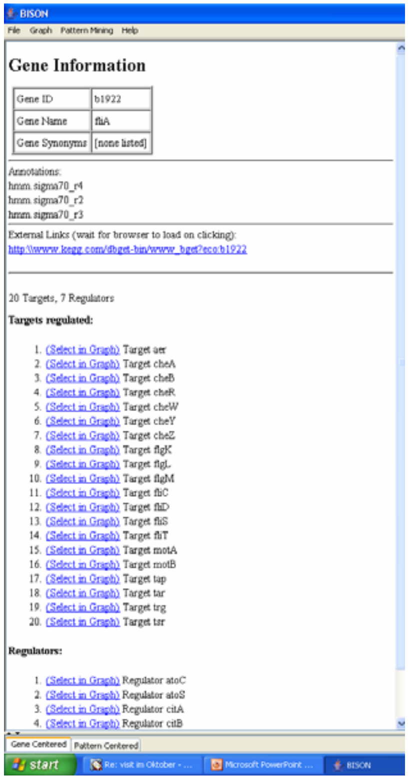

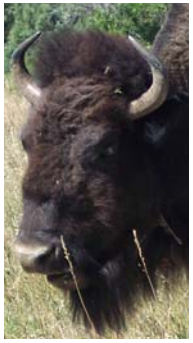

Pattern-centered analysis

- Select the ‘Patterns’ tab
- Use ‘Filter Patterns’ option to type in your property of interest. This can be a gene name, an HMM or other property (PF, GO). Click ‘Filter Patterns’

- Top left screen: the object information page will be the gene information page
- Top right screen: network visualization page
- Bottom screen: navigation page

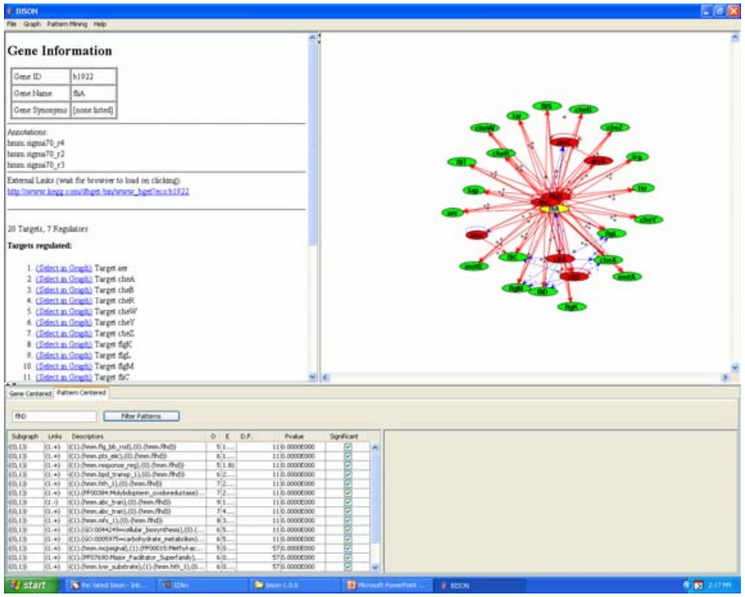

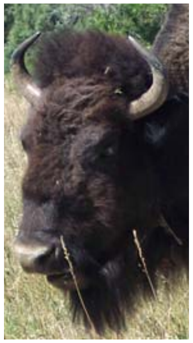

Navigation page (I)

- The ‘Descriptor’ column lists properties that are found in your gene, as well as in the genes that your gene product regulates:
  - (0) indicates properties found in the regulator
  - (1) indicates properties found in the regulated genes

- The ‘Links’ column indicates the regulation:
  - (+) positive regulation
  - (-) negative regulation

- The *p*-values are from the Chi-squared test

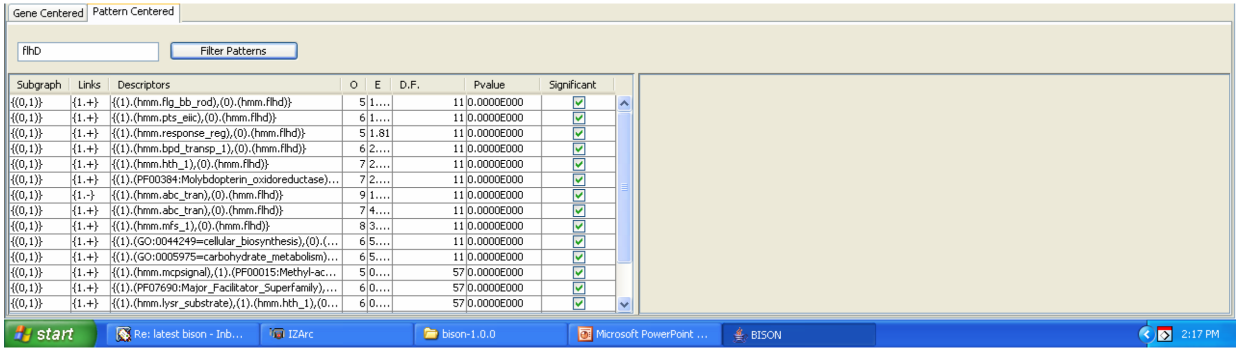

Subgraph	Links	Descriptors	O	E	D.F.	Pvalue	Significant
{(0,1)}	{1.+}	{(1).(hmm.flg_bb_rod),(0).(hmm.flhd)}	5	1....	11	0.0000E000	☑
{(0,1)}	{1.+}	{(1).(hmm.pts_eiic),(0).(hmm.flhd)}	6	1....	11	0.0000E000	☑
{(0,1)}	{1.+}	{(1).(hmm.response_reg),(0).(hmm.flhd)}	5	1.81	11	0.0000E000	☑
{(0,1)}	{1.+}	{(1).(hmm.bpd_transp_1),(0).(hmm.flhd)}	6	2....	11	0.0000E000	☑
{(0,1)}	{1.+}	{(1).(hmm.hth_1),(0).(hmm.flhd)}	7	2....	11	0.0000E000	☑
{(0,1)}	{1.+}	{(1).(PF00384:Molybdopterin_oxidoreductase)...	7	2....	11	0.0000E000	☑
{(0,1)}	{1.-}	{(1).(hmm.abc_tran),(0).(hmm.flhd)}	9	1....	11	0.0000E000	☑
{(0,1)}	{1.+}	{(1).(hmm.abc_tran),(0).(hmm.flhd)}	7	4....	11	0.0000E000	☑
{(0,1)}	{1.+}	{(1).(hmm.mfs_1),(0).(hmm.flhd)}	8	3....	11	0.0000E000	☑
{(0,1)}	{1.+}	{(1).(GO:0044249=cellular_biosynthesis),(0).(...	6	5....	11	0.0000E000	☑
{(0,1)}	{1.+}	{(1).(GO:0005975=carbohydrate_metabolism)...	6	5....	11	0.0000E000	☑
{(0,1)}	{1.+}	{(1).(hmm.mcpsignal),(1).(PF00015:Methyl-ac...	5	0....	57	0.0000E000	☑
{(0,1)}	{1.+}	{(1).(PF07690:Major_Facilitator_Superfamily),...	6	0....	57	0.0000E000	☑
{(0,1)}	{1.+}	{(1).(hmm.lysr_substrate),(1).(hmm.hth_1),(0...	6	0....	57	0.0000E000	☑

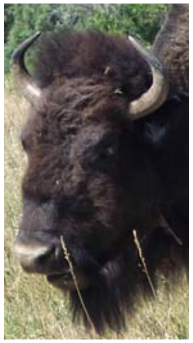

Navigation page (II)

- Select a line from the ‘Descriptor’ column

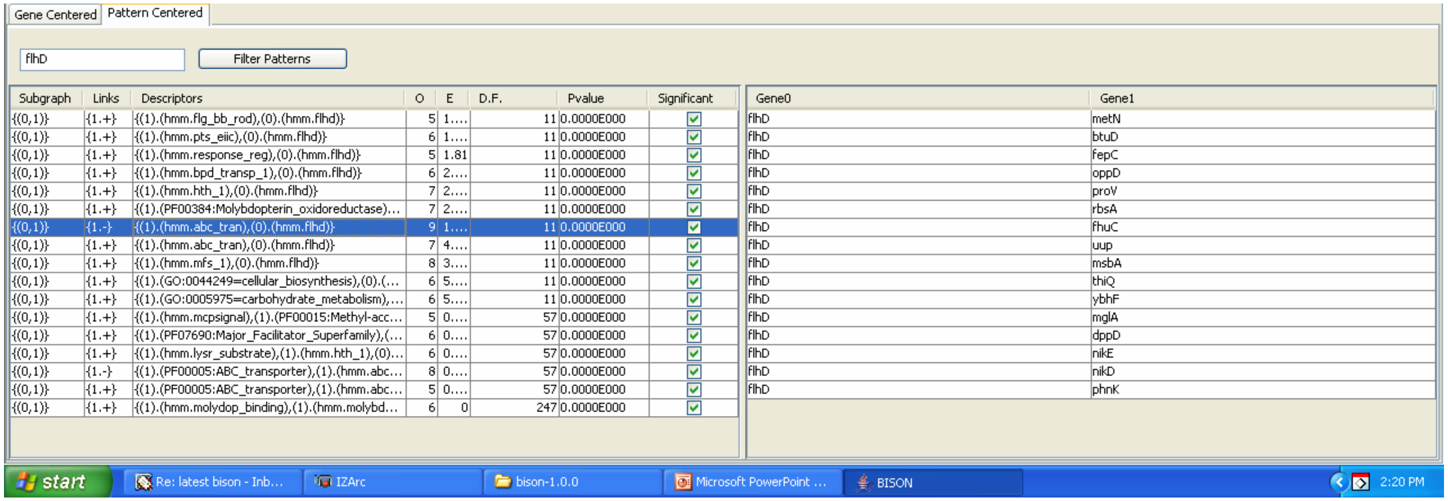

Subgraph	Links	Descriptors	O	E	D.F.	Pvalue	Significant
{(0,1)}	{1.+}	{(1).(hmm.flg_bb_rod),(0).(hmm.flhd)}	5	1.…	11	0.0000E000	☑
{(0,1)}	{1.+}	{(1).(hmm.pts_eiic),(0).(hmm.flhd)}	6	1.…	11	0.0000E000	☑
{(0,1)}	{1.+}	{(1).(hmm.response_reg),(0).(hmm.flhd)}	5	1.81	11	0.0000E000	☑
{(0,1)}	{1.+}	{(1).(hmm.bpd_transp_1),(0).(hmm.flhd)}	6	2.…	11	0.0000E000	☑
{(0,1)}	{1.+}	{(1).(hmm.hth_1),(0).(hmm.flhd)}	7	2.…	11	0.0000E000	☑
{(0,1)}	{1.+}	{(1).(PF00384:Molybdopterin_oxidoreductase)…	7	2.…	11	0.0000E000	☑
{(0,1)}	{1.-}	{(1).(hmm.abc_tran),(0).(hmm.flhd)}	9	1.…	11	0.0000E000	☑
{(0,1)}	{1.+}	{(1).(hmm.abc_tran),(0).(hmm.flhd)}	7	4.…	11	0.0000E000	☑
{(0,1)}	{1.+}	{(1).(hmm.mfs_1),(0).(hmm.flhd)}	8	3.…	11	0.0000E000	☑
{(0,1)}	{1.+}	{(1).(GO:0044249=cellular_biosynthesis),(0).(…	6	5.…	11	0.0000E000	☑
{(0,1)}	{1.+}	{(1).(GO:0005975=carbohydrate_metabolism),…	6	5.…	11	0.0000E000	☑
{(0,1)}	{1.+}	{(1).(hmm.mcpsignal),(1).(PF00015:Methyl-acc…	5	0.…	57	0.0000E000	☑
{(0,1)}	{1.+}	{(1).(PF07690:Major_Facilitator_Superfamily),(…	6	0.…	57	0.0000E000	☑
{(0,1)}	{1.+}	{(1).(hmm.lysr_substrate),(1).(hmm.hth_1),(0)…	6	0.…	57	0.0000E000	☑
{(0,1)}	{1.-}	{(1).(PF00005:ABC_transporter),(1).(hmm.abc…	8	0.…	57	0.0000E000	☑
{(0,1)}	{1.+}	{(1).(PF00005:ABC_transporter),(1).(hmm.abc…	5	0.…	57	0.0000E000	☑
{(0,1)}	{1.+}	{(1).(hmm.molydop_binding),(1).(hmm.molybd…	6	0	247	0.0000E000	☑

Gene0	Gene1
flhD	metN
flhD	btuD
flhD	fepC
flhD	oppD
flhD	proV
flhD	rbsA
flhD	fhuC
flhD	uup
flhD	msbA
flhD	thiQ
flhD	ybhF
flhD	mglA
flhD	dppD
flhD	nikE
flhD	nikD
flhD	phnK

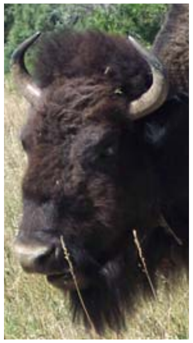

Navigation page (III)

- The right portion of the navigation page now contains two gene lists:
  - Gene 0: your gene of interest, encodes regulator
  - Gene 1: all the genes that are regulated by your regulator and whose encoded proteins contain the property that is listed in the selected line of the 'Descriptor' column and indicated with (1). This combination of properties is blue on the last slide.

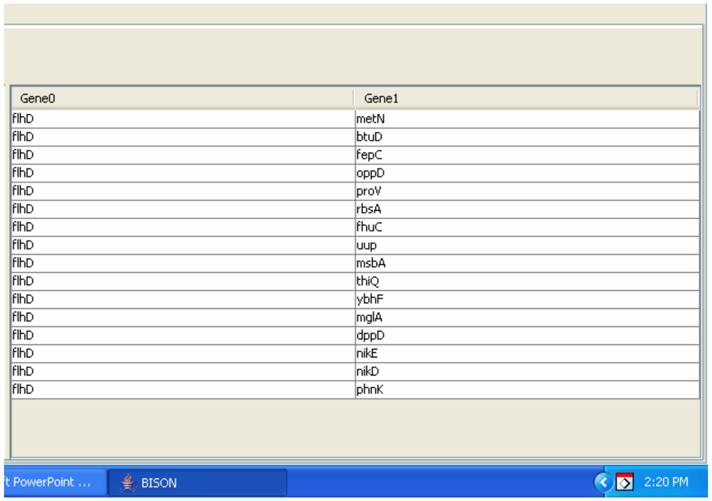

Gene0	Gene1
flhD	metN
flhD	btuD
flhD	fepC
flhD	oppD
flhD	proV
flhD	rbsA
flhD	fhuC
flhD	uup
flhD	msbA
flhD	thiQ
flhD	ybhF
flhD	mglA
flhD	dppD
flhD	nikE
flhD	nikD
flhD	phnK

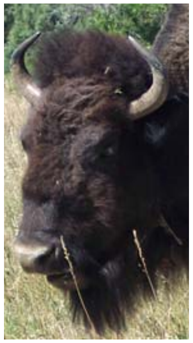

Pattern information page

- Select a line from the ‘Descriptor’ column
- The object information page will be the pattern information page. It indicates the patterns involved in this regulation (we suggest you resize the pages to get the best view at the pattern information page):
  - Gene 0: selected gene, encodes regulator
  - Descriptors 0: properties found in the regulator
  - Gene 1: target genes of the regulator
  - Descriptors 1: properties found in the proteins that are encoded by the regulated genes

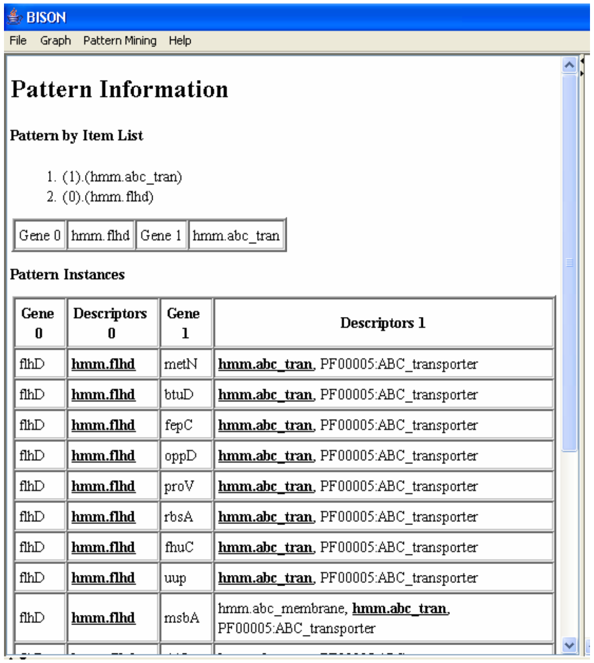

Pattern Information

Pattern by Item List

1. (1).(hmm.abc_tran)
2. (0).(hmm.flhd)

Gene 0	hmm.flhd	Gene 1	hmm.abc_tran

Pattern Instances

Gene 0	Descriptors 0	Gene 1	Descriptors 1
flhD	**hmm.flhd**	metN	**hmm.abc_tran**, PF00005:ABC_transporter
flhD	**hmm.flhd**	btuD	**hmm.abc_tran**, PF00005:ABC_transporter
flhD	**hmm.flhd**	fepC	**hmm.abc_tran**, PF00005:ABC_transporter
flhD	**hmm.flhd**	oppD	**hmm.abc_tran**, PF00005:ABC_transporter
flhD	**hmm.flhd**	proV	**hmm.abc_tran**, PF00005:ABC_transporter
flhD	**hmm.flhd**	rbsA	**hmm.abc_tran**, PF00005:ABC_transporter
flhD	**hmm.flhd**	fhuC	**hmm.abc_tran**, PF00005:ABC_transporter
flhD	**hmm.flhd**	uup	**hmm.abc_tran**, PF00005:ABC_transporter
flhD	**hmm.flhd**	msbA	hmm.abc_membrane, **hmm.abc_tran**, PF00005:ABC_transporter

You can select the Table and use ‘CTRL C’ to copy the table into a Microsoft Office document.

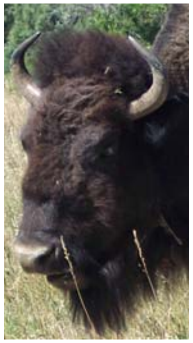

Network visualization page

- Select a line in the 'Gene' column of the navigation page
- The network visualization page will re-arrange around this new gene
- The object information page will switch to the gene information page and provide you with the information for your newly selected gene

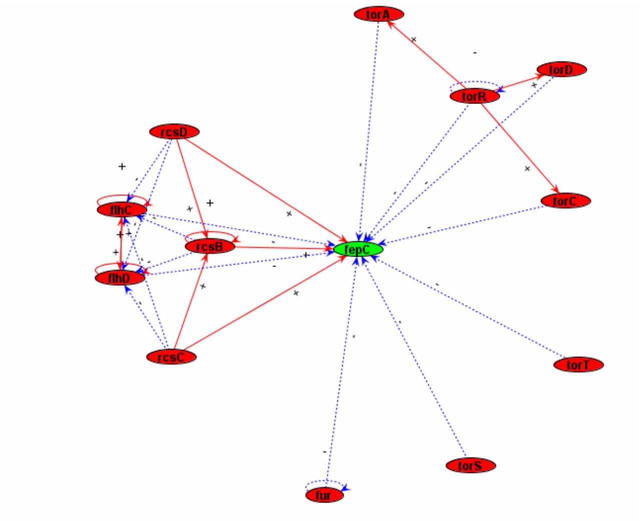

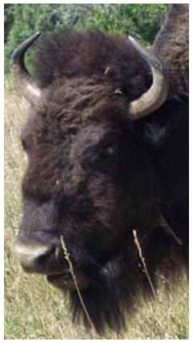

Your own data

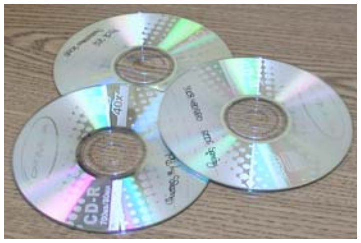

Your own data

- In addition to the network that is provided with BISON, you can add your own data
- Download detailed instructions from:
  - http://denton.cs.ndsu.nodak.edu/bison/

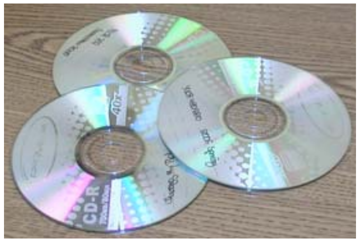

Adding a microarray experiment

- If you just want to add your own microarray experiment with *E. coli* K-12 and analyze it in the context of the network, add another network file (*.net) to the default_data directory in the following format (the first column is the regulator gene, the second the regulated gene). You can collect the data in Excel, save it as a tab delimited txt file and change .txt to .net manually:

b1892	b0019	-
b1892	b0020	-
b1892	b0030	+
b1892	b0032	+
b1892	b0033	+
b1892	b0036	+
b1892	b0037	+
b1892	b0059	+
b1892	b0064	+
b1892	b0066	+
b1892	b0069	+
b1892	b0070	+

- Add the name of your *.net file to the bison.config file

- Then load the default_data directory into BISON

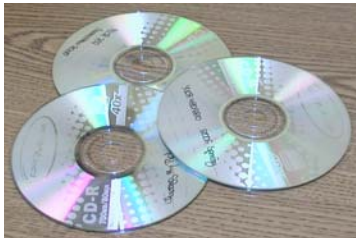

Adding a new network to BISON

- If you want to add a whole other organism:

- Create a new directory (New_data)

- The new data directory will need five data files (see page 8)
  - Entity file: lists each object (node) in the network
  - Alias file: lists the names each object should be known by
  - Synonym file: lists additional names objects may be known by
  - Pattern file: contains information gathered by pattern mining routines
  - Network file: lists the edges in the network

- The new data directory will also need two configuration files (see page 7)
  - Edgecolor file:colors edges in the network
  - Configuration file: lists all the files the network builds upon

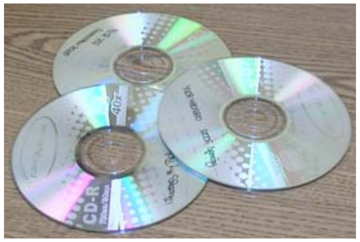

Entity file

- Lists each node that may be in the network

b0001 GO:0009308=amine_metabolism GO:0044249=cellular_biosynthesis GO:0006519=amino_acid_and_derivative_metabolism GO:0044271=nitrogen_compound_biosynthesis GO:0006082=organic_acid_metabolism

b0002 GO:0016301=kinase_activity GO:0019538=protein_metabolism GO:0016774=phosphotransferase_activity,_carboxyl_group_as_acceptor hmm.homoserine_dh GO:0000287=magnesium_ion_binding hmm.nad_binding_3 GO:0003959=NADPH_dehydrogenase_activity GO:0044271=nitrogen_compound_biosynthesis GO:0006790=sulfur_metabolism GO:0030554=adenyl_nucleotide_binding GO:0009308=amine_metabolism GO:0006519=amino_acid_and_derivative_metabolism GO:0044249=cellular_biosynthesis hmm.aa_kinase GO:0016616=oxidoreductase_activity,_acting_on_the_CH-OH_group_of_donors,_NAD_or_NADP_as_acceptor PF00696:Amino_acid_kinase_family GO:0006082=organic_acid_metabolism

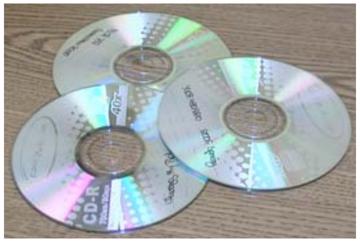

Alias file

- Lists each node from the entify file with the name that should be associated with it

b0001	*thrL*
b0002	*thrA*
b0003	*thrB*
b0004	*thrC*
b0005	*yaaX*
b0006	*yaaA*
b0007	*yaaJ*
b0008	*talB*
b0009	*mog*
b0010	*yaaH*

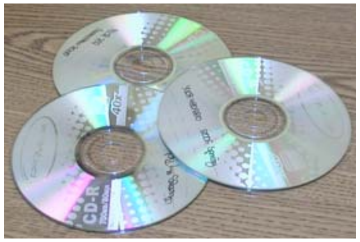

Synonym file

- Lists nodes from the entity and alias file that have names associated with them in addition to the alias

b0116	*lpdA*
b0161	*htrA*
b0178	*skp*
b0591	*ybdA*
b0755	*pgmA*
b1009	*ycdJ*
b1136	*icdA*

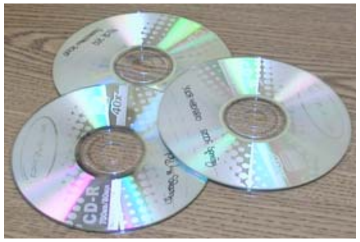

Pattern file

- In order to create this file, you will have to run the pattern mining engine of BISON (see page 18)

- Lists patterns found with the pattern mining library

{(0).(hmm.hatpase_c),(0).(hmm.hiska),(1).(PF00384:Molybdopterin_oxidoreductase),(1).(hmm.molybdopterin),(1).(hmm.molydop_binding)} 5 0.821119550753283 4.6117 1016 0 PASS

{(0,1)} {1.+}

b3404 b0894

b3404 b1224

b3404 b1468

b3911 b1468

b3911 b1224

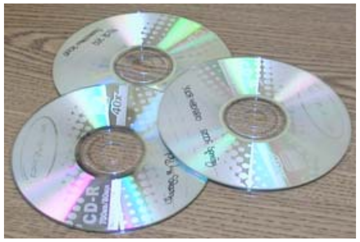

Network file

- Lists node pairs from the entity file that form edges in the network

b0020	b0019	+
b0020	b1482	+
b0034	b0035	+
b0034	b0036	+
b0034	b0037	+
b0034	b0038	+
b0034	b0039	+
b0034	b0040	+
b0034	b0041	+
b0034	b0042	+
b0034	b0043	+
b0034	b0044	+
b0064	b0061	+
b0064	b0061	-
b0064	b0062	+
b0064	b0062	-
b0064	b0063	+
b0064	b0063	-

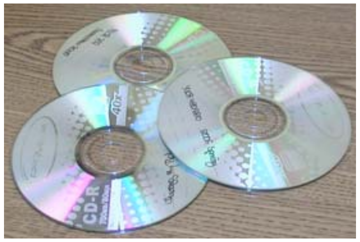

Configuration file

- Determines how to color edges on the networks

LABEL	RED_VALUE	GREEN_VALUE	BLUE_VALUE	ALPHA	DOTTED/SOLID
+	1f	0f	0f	1f	SOLID
-	0f	0f	1f	1f	DOTTED
+-	1f	1f	0f	1f	SOLID

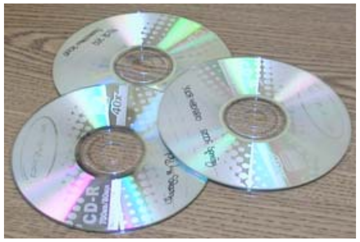

Bison.config details

- Your new data directory must contain a BISON configuration file (note that these files will be replaced by your files):

  # "E.coli" from RegulonDB and Dr. Pruess data with annotations from Pfam and Wisconsin GENE ID

  ENTITYFILE ecoli_entity.txt

  ALIASFILE ecoli_alias.txt

  SYNONYMFILE ecoli_syn.txt

  PATTERNFILE patterns.out

  NETWORKFILE flhD_microarray.net edgeColors.txt

  NETWORKFILE regulon.net edgeColors.txt

  NETWORKFILE pruess.net edgeColors.txt

  NETWORKFILE 2component.net edgeColors.txt

  LINK http:\\www.kegg.com/dbget-bin/www_bget?eco: ID

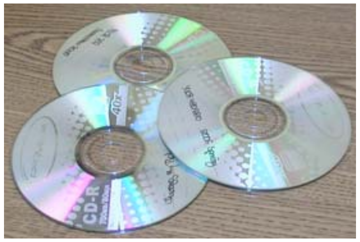

Opening new data

- Click ‘Bison.exe’
- Click ‘File’, select ‘Load File Directory’ and select ‘New_Data’

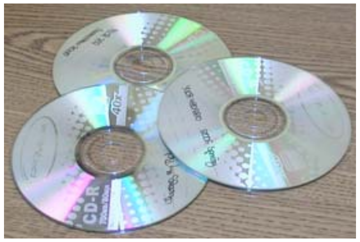

Reference

Besemann, C., Denton, A., Carr, N.J., and Prüß, B.M. BISON: A Bio-Interface for the Semi-global analysis Of Network patterns. 2006. Source Code for Biology and Medicine, Volume 1.

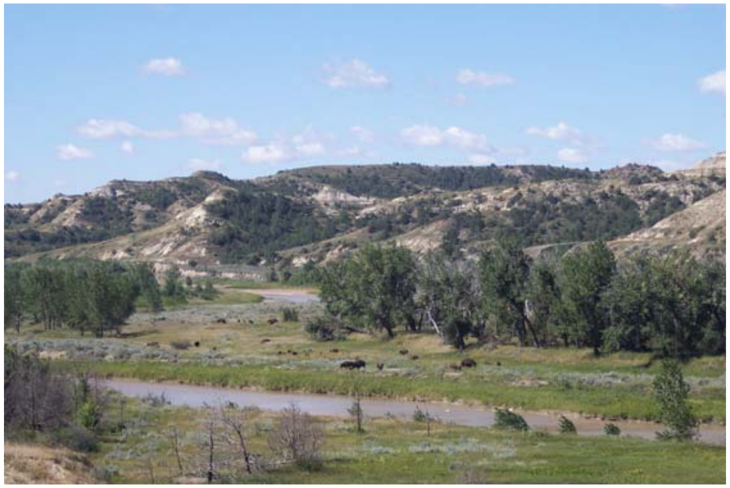

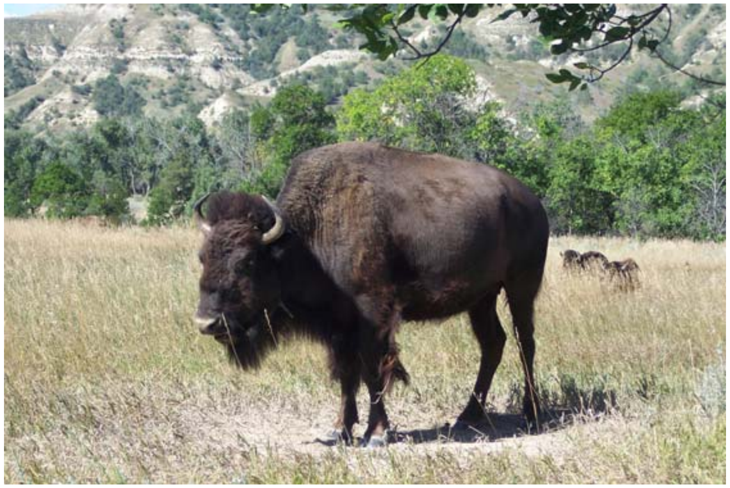

Please, reference this paper when using BISON
